# Estimating the spatial distribution of evapotranspiration within the Pra River Basin of Ghana

**DOI:** 10.1016/j.heliyon.2021.e06828

**Published:** 2021-04-21

**Authors:** J.J. Nsiah, C. Gyamfi, G.K. Anornu, S.N. Odai

**Affiliations:** a Department of Civil Engineering, Faculty of Civil and Geo Engineering, Kwame Nkrumah University of Science and Technology (KNUST), Kumasi, Ghana; b Department of Civil Engineering, Faculty of Engineering, Takoradi Technical University (TTU), Takoradi, Ghana; c Regional Water and Environmental Sanitation Centre, Kumasi (RWESCK), KNUST, Ghana; d Office of the Vice Chancellor, Accra Technical University, Accra, Ghana

**Keywords:** Evapotranspiration, SEBAL model, Spatial variation, Pixels, Remote sensing and satellite imagery

## Abstract

It is important in water resource planning to accurately estimate the spatial distribution of evapotranspiration (ET) as an input parameter for hydrological studies. Although, conventional pan evaporation, lysimetric and eddy covariance techniques have been used, they only estimate point values. Hence, this study aimed at estimating the spatial distribution of ET within the Pra River Basin (a forest ecological zone) of Ghana, using cloud-free Landsat 8 (OLI/TIRS) satellite images employing the SEBAL methodology. The study further estimates the spatial distribution ET in relation to major climatic variables, Land Use Land Cover (LULC) types and energy balance components. The overall spatial distribution of ET had a mean value of 5.63 mm/day. Spatial distribution of ET (mm/day) for water body (5.51–7.81) and uncultivated forest (5.10–7.71) were high, while moderately average values were observed for logged forest (4.80–7.51). Settlement and bare landscapes observed low rates ((2.05–5.10) mm/day). Spatially, ET was higher in the upper western, central and the eastern parts of the basin, but lower in the northern part and pockets of areas at the southern part of the basin where settlement/bare landscape and logged forest dominate. Areas with high temperature and high solar radiation experiences high ET, while low wind speed, low to average temperature and solar radiation areas experience low ET. Also, areas with both high net radiation and ground heat flux but low to average sensible heat flux experiences high ET and vice versa. Linear regression analysis showed good fit with slope of 0.76 and R^2^ of 0.93 indicating that 93 % of the variations in observed field measurement of ET fitted perfectly well with ET distributions generated by the SEBAL model.

## 1 Introduction

It is crucial and important in water resource planning to accurately estimate the spatial distribution of evapotranspiration (ET) as an input parameter to simulate and predict both present and future hydrological processes of major river basins (Kundu et al, 2017, 2018; Long and Singh, 2012; Oguntunde, 2004; Bastiaanssen et al., 2005). ET is the gaseous component of the water cycle that returns about 60 % of global precipitation to the atmosphere (Andreini et al., 2000; Oki and Kanae, 2006). Considering major river basins in West Africa such as the Volta, ET is reported to account for approximately 90% of total catchment rainfall (Andreini et al., 2000). Thus, ET is the most influential component of the water cycle with immense research potential in hydrological studies (Andam-Akorful et al., 2015; Bonemberger et al., 2018; Opoku-Duah, 2007 and Sett et al., 2018). In view of this, accurate estimation of ET at both local and regional scales remains a fundamental tool for water resources planning and management (Allen et al., 2007; Kundu et al., 2018; Li et al., 2018). Intriguingly, ET is the most complex hydrologic parameter to estimate (Bastiaanssen et al., 1998; Sett et al., 2018) as it depends on factors such land use land cover (LULC) and climate change (Li et al., 2017) whose dominance is influenced by anthropogenic activities (Awotwi et al., 2019). ET is highly affected by LULC properties such as Normalized Difference Vegetation Index (NDVI) and Leaf Area Index (LAI) which collectively express the percentage of leaf area covering the land to the total area of cultivated land. NDVI and LAI can be derived from satellite spectral reflectance and radiance (Jafaar and Ahmad, 2018; Gao and Zhang, 2006). Also, the effects of climate change on the spatial distribution of ET has been studied (Burn and Hesch, 2007; Dinpashoh et al., 2011; Gao and Zhang, 2006; and Zhang et al., 2011) with diverse conclusive remarks. For example, Gao and Zhang (2006) and Zhang et al. (2011) concluded that ET has decreased in most countries, and that the decrease might be caused by a reduction in solar radiation and a decrease in wind speed. While other researchers (Burn and Hesch, 2007; Dinpashoh et al., 2011) have opined that ET increases in some areas were mainly related to increase in wind speed. In areas where higher net solar radiation is recorded, air surface temperature tends to increase leading to higher ET. Thus, accurate estimation of the spatial distribution of ET has to consider the involvement of major climatic factors and the land cover features controlling the hydrologic processes within the basin. Methods of estimating ET include empirical methods, conventional field measurement and in recent times, satellite derived remote sensing methods. Empirical mathematical models such as FAO modified Penman Monteith, Makkink, Blaney Criddle, Hargreaves and Priestly-Taylor methods are normally employed to compare field estimates of ET. Conventional field methods such as pan evaporation, lysimetric and eddy covariance techniques only estimate point source values and so fail to estimate the spatial distribution of this important hydrologic parameter (Bastiaanssen et al., 1998, 2005; Mkhwanazi et al., 2015; Rahimsadegan and Janani, 2019; Snyder et al., 2005 and Zhang et al., 2011). For water resources planning on a large scale, point source measurement of ET will be impracticable due to constrains of time, human and financial resources. To overcome this challenge, the application of Geographic Information Systems (GIS) and Remote Sensing (RS) techniques in deriving the spatial distribution of continuous data for ET using surface energy balance techniques has been explored (Allen et al., 2007; Bastiaanssen et al., 2005; Liou and Kar, 2014; Li and Zhao, 2010a, b; Mkhwanazi et al., 2015; and Nouri et al., 2017). These GIS and RS techniques employ multi-spectral bands from satellite images to provide spatial information on ET over many regions (Ayad et al., 2016). The various methods that have evolved from this technique include the Surface Energy Balance Algorithm for Land (SEBAL) (Bastiaanssen et al., 1998), Mapping ET at High Resolution with Internalised Calibration (METRIC) (Allen et al., 2007), and the Simplified Surface Energy Balance (Su, 2002) etc. Amongst these methods, SEBAL is the most widely used model as it provides a robust and efficient tool for estimating the spatial distribution of ET (Bastiaanssen, 2000; Bastiaanssen et al., 2005; Jaber et al., 2016; Li et al., 2018; and Yonggwan and Seongjoon, 2016). The most unique and innovative component of this model lies in its use of near-surface temperature gradient, dT, indexed to the radiometric surface temperature, Ts, to eliminate the need for absolute surface aerodynamic temperature calibration (Allen et al., 2007). This in-built calibration reduces the impact of aerodynamic resistance to vapour transport on the spatial distribution of ET. The overall accuracy of ET from SEBAL is around +/−15% (Ayad et al., 2016). The accuracy of the model can be estimated using simple linear regression model to identify and characterize the relationship between observed field measurement of spatial distribution of ET and that of the model. While a correlation coefficient can be calculated as a measure of the strength of the monotonic relationship between the observed and modelled ET distributions. Luckily, the model has been used and implemented successfully in numerous studies in different countries (Hendrickx et al., 2006; Sun et al., 2011). In Ghana, only a few studies have tested the use of the SEBAL model in estimating ET with majority of these works focusing on the Volta Basin. Compaoré et al. (2008) mapped ET in the White Volta sub-basin during dry season using Landsat and MODIS images, and deduced that SEBAL had potential for mapping ET over tropical areas. In another study, Opoku-Duah et al. (2008) identified that ET estimates from MODIS driven by SEBAL under-performed by up to 2mm/day when compared with that obtained from the Penman-Monteith and eddy covariance methods and attributed the inconsistency to spatial mismatch. All these previous studies undeniably prove that SEBAL algorithm is capable of estimating the spatial and temporal distribution of ET especially, in areas with poor and/or inadequate data such as Ghana and other African countries. However, this SEBAL model has not been studied in the different covers and scales within the tropical forest zone of southern Ghana. Moreover, studies using RS in estimation of ET have not considered the effect of major climatic factors and LULC on spatial distribution of ET. Therefore, this study aimed at estimating the spatial distribution of ET using SEBAL algorithm and cloud-free Landsat 8 (OLI/TIRS) satellite imagery within the Pra River Basin (PRB) of Ghana. It also seeks to assess the distribution of ET in relation to major climatic variables (temperature, solar radiation and wind speed), LULC types and energy balance components. This research will present relevant information on ET for the estimation of both present and future water use for irrigation agriculture, water balance components and drivers of climate change and variability within the PRB.

## 2 Study area

**Figure 1:**
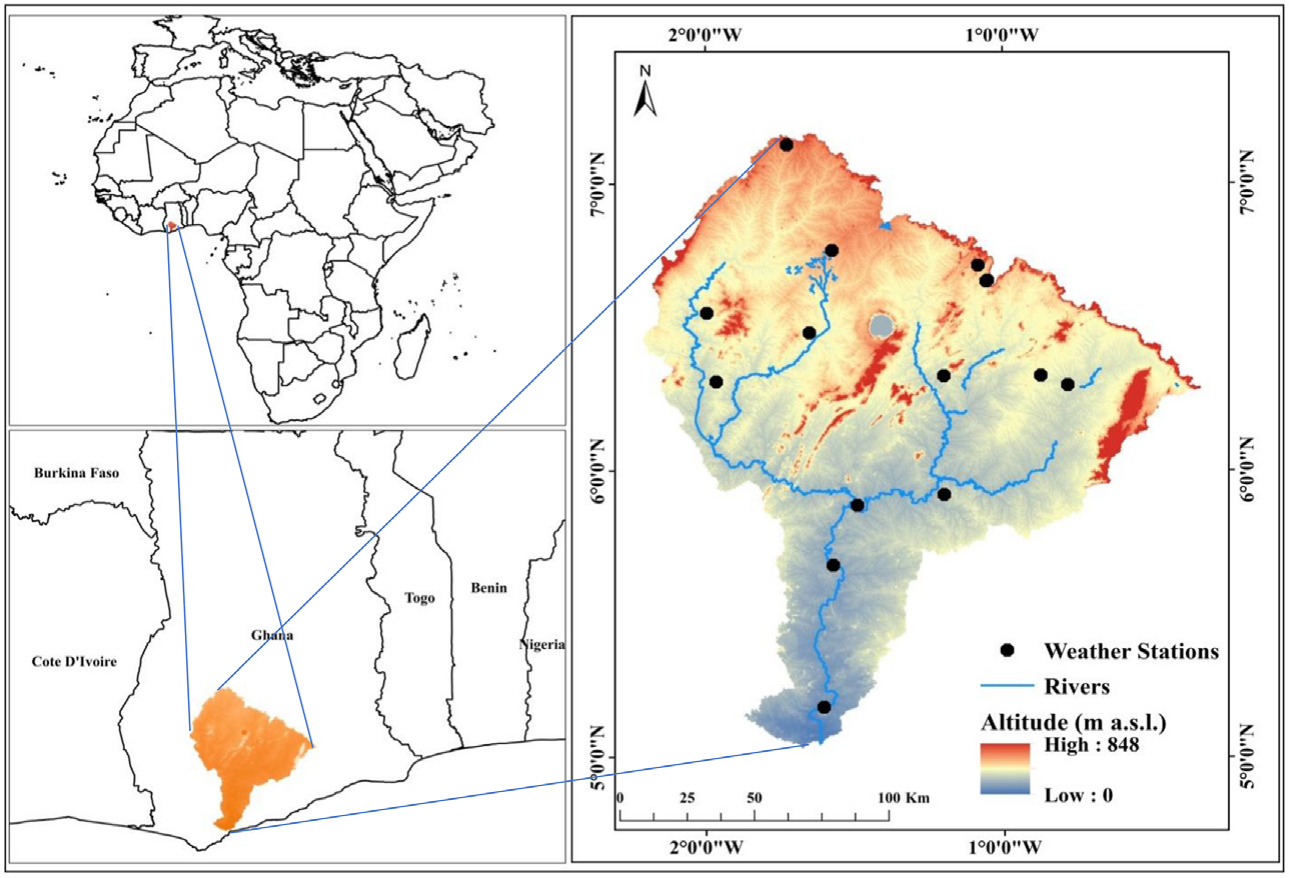
Location, DEM, weather stations and river flow patterns of the PRB.

The PRB (Figure 1) with a drainage area of 22,106 km^2^, located within the forest ecological zone of Ghana lies between latitudes 4^0^49^’^23″ N - 7^0^13^’^1″ N and longitudes 0^0^11^’^56″ W - 2^0^58^’^48″ W. The elevation of the basin ranges between 0 and 848 m with an average of 200 m. The climate is considered to be tropical monsoon climate (Am), according to the updated Köppen-Geiger climate classification by Kottek et al. (2006). It has two rainy seasons spanning from April–June and September–November with mean annual rainfall of about 1,600 mm. Air temperature increases toward the northern part of the basin with average minimum and maximum rates of 21 °C and 32 °C respectively. The PRB constitutes a major source of water supply with about 48 % being used for irrigation agriculture. The basin also hosts the largest cocoa plantations in Ghana. Other economic activities include oil palm plantation and the cultivation of varieties of food crops.

## 3 Data and methods

Three (3) cloud-free Landsat 8 (OLI/TIRS) images (row 193 path 56, row 194 path 56 and row 194 path 55) with DOY/YEAR 31/2018 and ASTER DEM were downloaded from the United States Geological Survey website (https://earthexplorer.usgs.gov/). The images were atmospherically corrected, geometrically rectified and radiometrically calibrated prior to being mosaicked and masked to obtain an accurately referenced and well-defined image covering the spatial extent of the PRB. The image pre-processing was done using ArcGIS 10.4.1 and ERDAS IMAGINE software. In classifying the images, ground truth points were collected from field for accuracy assessment by employing the random forest algorithm. The error matrix was used to assess the accuracy of the classification. While the probability of classification and the overall accuracy (OA) in the error matrix computed and the kappa coefficient determined. Meteorological data (temperature, solar radiation, wind speed, relative humidity, precipitation and pan evaporation data) from fifteen weather stations (Figure 1) acquired from the Ghana Meteorological Agency was used.

### 3.1 The SEBAL model

The SEBAL model is an image processing model that estimates the ET flux for each pixel of the image by solving the terms of the surface energy balance equation derived from the visible, NIR and thermal-IR bands of the electromagnetic spectrum (Allen et al, 2002, 2007; Bastiaanssen et al., 2005; Sun et al., 2011). SEBAL computes net radiation (R_n_), sensible heat flux (H) and soil heat flux (G) for every pixel and the latent heat flux (LE) deduced as a residual of the surface energy budget Eq. (1) expressed as:

(1) $$\lambda ET=R_{n}-G-H$$

where; λ*ET* is the latent heat (W/m^2^), *R*_*n*_ is the net radiation flux at the surface (W/m^2^), *G* is the soil heat flux (W/m^2^), and *H* is the sensible heat flux to the air (W/m^2^). The net radiation (*R*_*n*_) was computed by estimating the algebraic difference between all outgoing and incoming radiant energy fluxes expressed by the surface radiation balance Eq. (2).

(2) $$R_{n}=\left(1-\alpha \right)\times R_{S↓}+R_{L↓}-R_{L↑}-\left(1-\varepsilon_{0}\right)\times R_{L↓}$$

where; *R*_*S↓*_ is incoming shortwave radiation (Wm^−2^), *R*_*L↓*_is incoming longwave radiation (Wm^−2^), R_L↑_ is outgoing longwave radiation (Wm^−2^); $\varepsilon_{0}$ is surface thermal emissivity [-]; α is surface albedo [-]. The soil heat flux (*G*) was deduced following an empirical Eq. (3) developed by Bastiaanssen (2000) representing values near midday given as;

(3) GRn=Tsα(0.0038α+0.0074α2)(1−0.98NDVI4)

where; T_S_ is land surface temperature (^O^C), α is the albedo [-] and NDVI is Normalized Difference Vegetation Index [-]. NDVI is a very sensitive parameter indicating the ratio of the differences in reflectivity for the NIR and RED bands to their sum expressed in Eq. (4) as:

(4) $$NDVI=\frac{\left(\rho_{5}-\rho_{4}\right)}{\left(\rho_{5}+\rho_{4}\right)}$$

where; ρ_4_ and ρ_5_ are the reflectivities for bands 4 and 5 respectively. Values for NDVI range between -1 and +1. Green surfaces have a NDVI between 0 - 1 and water and clouds are usually less than zero. NDVI is thus, a measure of the amount and condition of green vegetation. The sensible heat flux (H) which represents heat loss to the air by convection and conduction owing to temperature difference was also computed from Eq. (5).

(5) $$H=\rho Cp\frac{dT}{r_{ah}}$$

where; *H* is sensible heat flux (Wm^−2^); *ρ* is density of air (kg/m^3^); *Cp* is specific heat capacity of air (1004 J/kg/K); *dT* is near surface temperature difference (T_1_ – T_2_) (K) between two heights (Z_1_ and Z_2_) (m) and *r*_*ah*_ is aerodynamic resistance to heat transport (s/m).

### 3.2 SEBAL toolbox development

With reference to the SEBAL manual described by Allen et al. (2002), a spatial analyst toolbox consisting of 3 sub-models was designed and built with the model builder in ArcGIS 10.4.1 to compute the spatial distribution of ET. These sub-models compute (i) land surface temperature, (ii) iterate to compute sensible heat flux (H) from near surface temperature difference (dT) and the aerodynamic resistance to vapour transport (*r*_*ah*_) and (iii) latent heat of vaporisation (λET) as residual of the net radiation (R_n_), soil heat flux (G) and sensible heat flux (H).

#### 3.2.1 Land surface temperature

The land surface temperature was computed by first converting the digital numbers of the pixels of the thermal infra-red (TIR) bands into Top of Atmosphere (TOA) spectral radiance using rescaling factors given in the metadata of the satellite imagery according to Eq. (6).

(6) $$L\lambda =ML\times Q_{cal}+AL$$

where; Lλ is TOA spectral radiance (Watts/(m^2^/srad/μm)), ML is band-specific multiplicative rescaling factor from the metadata given as: RADIANCE_MULT_BAND_x, where x is the band number), AL is band-specific additive rescaling factor from the metadata (RADIANCE_ADD_BAND_x), and Q_cal_ is Quantized and calibrated standard product pixel values (DN). The top of planetary reflectance for each band based on spectral radiance at the sensor aperture was computed by Eq. (7) given by Allen et al. (2002).

(7) $$\rho_{\lambda }=\frac{\pi \times L_{\lambda }}{ESUN_{\lambda }\times \cos\;\theta \times d_{r}}$$

where; $\rho_{\lambda }$ is TOA planetary reflectance for each band, $d_{r}$ is the inverse squared relative Earth-sun distance in astronomical terms [-], ESUN_λ_ is mean solar exo-atmospheric spectral irradiance on a surface perpendicular to the sun's ray (W/m^2^/μm). Table 1 shows ESUN_λ_ values used in the study.

**Table 1:** Exo-atmospheric spectral solar irradiance (ESUN) of Landsat-8 OLI bands.

Band	Band 2	Band 3	Band 4	Band 5	Band 6	Band 7	Band 9
ESUN_λ_	2011.3	1853.3	1562.8	956.4	245	237.8	399.7

The solar zenith angle (cos θ) is computed using the header file data on sun elevation angle (β) where θ = (90^0^ - β). θ in decimal degrees is subsequently converted into radians. The inverse squared relative Earth-Sun distance in astronomical terms given as $d_{r}$ was also computed using Eq. (8).

(8) $$d_{r}=1+0.033\times \cos\left(Julian\_day\times \frac{2\pi }{365}\right)$$

The Earth-Sun distance ($d_{r}$) is then used in calculating the surface albedo according to the SEBAL Manual (Allen et al., 2002), by first calculating the top of the atmosphere albedo computed using Eq. (9).

(9) $$\alpha_{toa}=\sum \left(\omega_{\lambda }\times \rho_{\lambda }\right)$$

Where ω_λ_ is a weighing coefficient for each band expressed in Eq. (10) as:

(10) $$\omega_{\lambda }=\frac{ESUN_{\lambda }}{\sum ESUN_{\lambda }}$$

The TOA albedo is then converted to surface albedo using Eq. (11) by Allen et al. (2002).

(11) $$\alpha =\frac{\left(\alpha_{toa}-\alpha_{path\_radiance}\right)}{\tau_{SW}^{2}}$$

where α is surface albedo; $\alpha_{path\_radiance}$ is the incoming shortwave radiation flux reflected back to the sensor (ranged from 0.025 to 0.04), in SEBAL the value of 0.03 is used. The atmospheric transmissivity ($\tau_{SW}$) is defined as the fraction of incident radiation that is transmitted by the atmosphere and it represents the effects of absorption and reflection occurring within the atmosphere. This was computed by the expression given Eq. (12).

(12) $$\tau_{SW}=0.75+2\times 10^{-5}\times z$$

where z is the elevation above mean sea level of the study area obtained from the DEM.

The next is to calculate the emissivity (ε_ΝΒ_) representing surface behaviour for thermal emission in the relatively narrow band 6 of Landsat (10.4–12.5 μm). This is also expressed by the following empirical Eqs. (13), (14), and (15), where NDVI >0:

(13) $$\varepsilon_{NB}=0.97+0.0033\times LAI$$

for LAI <3 and ε_NB_ = 0.98, when LAI≥3

The corrected thermal radiance from the surface (R_c_) is calculated following Eq. (14) by Weng et al. (2004).

(14) $$R_{C}=\frac{L_{6}-R_{P}}{\lambda_{NB}}-\left(1-\varepsilon_{NB}\right)R_{sky}$$

where; L_6_ is the spectral radiance of band 6 (W/m^2^/sr/μm), R_p_ is the path radiance in the .4–12.5 μm band (W/m^2^/sr/μm), R_sky_ is the narrow band downward thermal radiation for a clear sky (W/m^2^/sr/μm), and τ_NB_ is the narrow band transmissivity of air (10.4–12.5 μm). Units for R_c is_ W/m^2^/sr/μm. The corrected thermal radiance (R_c_) is the actual radiance emitted from the surface whereas L_6_ is the radiance that the satellite “sees”. Thus, the land surface temperature (T_S_) is then computed from the corrected thermal radiance (R_C_) as expressed in Eq. (15).

(15) $$T_{S}=\frac{K_{2}}{\ln\left(\frac{\varepsilon_{NB}\times K_{1}}{R_{C}}+1\right)}$$

where; R_C_ is the corrected thermal radiance from the surface, K_1_ is 774.89 W/m^2^/ster/μm, and K_2_ is 1321.08 in kelvin. L_λ_ is spectral radiance in W/m^2^/ster/μm and is calculated using Eq. (16).

(16) $$L_{\lambda }=0.0370588\times DN+3.2$$

#### 3.2.2 Sensible heat flux

The sensible heat flux (H) which represents heat loss to the air by convection and conduction owing to temperature difference is computed using Eq. (17).

(17) $$H=\rho Cp\frac{dT}{r_{ah}}$$

where; *H* is sensible heat flux (Wm^−2^); *ρ* is density of air (kg/m^3^); *Cp* is specific heat capacity. of air (1004 J/kg/K); *dT* is near surface temperature difference (T_1_ – T_2_) (K) between two heights (*z*_*1*_ and *z*_*2*_) (m) and *r*_*ah*_ is aerodynamic resistance to heat transport (s/m). Since the above equation is a function of three variables (temperature difference, surface roughness and wind speed) with two unknowns (*r*_*ah*_ and *dT*), the two anchor pixels named hot and cold pixels were used to facilitate the computation. The cold pixel representing a well-watered and fully-vegetated crop field with optimum ET was carefully selected from field investigation. The hot pixel which represents a bare dry agriculture field with an almost zero ET was similarly selected from careful field inspection (Allen et al., 2002). The aerodynamic resistance (*r*_*ah*_) was computed from Eq. (18) for neutral atmospheric stability conditions.

(18) $$r_{ah}=\frac{\ln\left(\frac{z_{2}}{z_{1}}\right)}{u*\times k}$$

where; *z*_*1*_ and *z*_*2*_ are heights (m) above zero plane displacement of vegetation, u∗ is the frictional velocity (m/s) quantifying turbulent velocity fluctuations in the air, and k is Von Karman's constant (0.41). The frictional velocity was estimated from field measurement of wind speed data using the logarithmic wind law Eq. (19) at neutral atmospheric conditions.

(19) $$u*=\frac{ku_{x}}{\ln\left(\frac{z_{x}}{z_{om}}\right)}$$

Where; *u*_*x*_ is the field wind speed (m/s) measurement at *z*_*x*_ is 2m above the surface and, *z*_*om*_ is *0.12h*. *z*_*om*_ is the momentum roughness length (m) which is a measure of the drag and skin friction for the air layer interacting with the surface. According to Allen et al. (2002), *h* is 0.3m. The frictional velocity at blending height (*u*_*200*_), where no effect from surface roughness is felt was further calculated from Eq. (20) using a rearranged form of Eq. (19).

(20) $$u_{200}=u*\frac{\ln\left(\frac{200}{z_{om}}\right)}{k}$$

The frictional velocity for each pixel was then calculated using Eq. (21).

(21) $$u_{pi}^{*}=\frac{ku_{200}}{\ln\left(\frac{200}{z_{om}}\right)}$$

where all parameters have their usual meaning. The *z*_*om*_ for each pixel was also obtained from the land-use map of the basin as proposed by Allen et al. (2002). Here, the assigned momentum roughness length for forest, logged forest, settlement and water are chosen from Table 2 below.

**Table 2:** Assign values of momentum roughness length to specific land use class.

No	Land use class	momentum roughness length (*z*_*om*_) [m]
1	Water	0.0005
2	Settlement	0.2
3	Forests	0.5
4	Logged forest	0.02

The model then establishes a linear relationship between *dT* and the surface temperature *Ts* estimated from the field measurement of temperature data using Eq. (22).

(22) $$dT=b+aT_{s}$$

where; *a* and *b* are the correlation coefficients. Values of *Ts*, *dT*_*cold*_ and *dT*_*hot*_ are estimated from the choice of values for the cold and hot pixels *H*_*cold*_ and *H*_*hot*_. A linear plot of *dT*_*cold*_ VS. *T*_*s_cold*_ and *dT*_*hot*_ VS. *T*_*s_hot*_ then determines the coefficients *a* and *b*. The *r*_*ah*_ for each pixel are then calculated using Eq. (18) with values of *z*_*1*_ = 0.1m and *z*_*2*_ = 2.0m to estimate the initial value of *H* through an iterative process using the Monin-Obukhov length formula by correcting for atmospheric instability due to buoyancy effect of surface heating until H stabilizes.

#### 3.2.3 Latent heat flux

The rate of latent heat loss from the surface due to evapotranspiration was finally computed for each pixel using the energy balance Eq. (6). The instantaneous ET represented by the equivalent water depth lost to the atmosphere in an hour is computed using Eq. (23).

(23) $$ET_{inst}=3600\frac{\lambda ET}{\lambda }$$

where; ET _inst_ is the instantaneous ET (mm/hr); 3600 is the time conversion from seconds to hours, and λ is the latent heat of vaporization (J/kg).The REF-ET (Reference Evapotranspiration) Calculation Software (Allen, 2000) was used to calculate the hourly ET_r_ values utilising wind speed, air temperature and relative humidity data for the DOY of the image. The evaporative fraction (ET_rF_) was computed from Eq. (24) as the ratio of instantaneous ET_inst_ to the reference ET_r_.

(24) $$E{T_{r}}_{F}=\frac{ET_{inst}}{ET_{r}}$$

Where; ET_inst_ is instantaneous ET (mm/hr) and ET_r_ is the reference ET at the time of the image from the REF-ET software (mm/hr). ETr_F_ is similar to the well-known crop coefficient, Kc. ETr_F_ is used to extrapolate ET from the image time to 24-hour period using Eq. (25).

(25) $$ET_{24}=E{T_{r}}_{F}\times ET_{r\_24}$$

where; ET_24_ is the estimated daily ET (mm/day); ET_r_24_ is the cumulative 24-hour ET for the day of the image. Due to the unavailability of direct ET flux measurement, recorded pan evaporation data from fifteen (15) meteorological stations within the basin were used to validate the SEBAL model for the day of satellite passage. The actual evapotranspiration was computed from the pan evaporation data using the crop coefficient approach expressed in Eq. (26).

(26) $$ET=K_{C}E_{pan}$$

where; ET is the estimated actual evapotranspiration evaporation (mm/day), E_pan_ is the measured pan evaporation data (mm/day), and K_C_ is the crop coefficient [-]. The crop coefficient was calculated according to Han and Zhen, (2004a, b).

## 4 Results and discussions

### 4.1 Land use land cover classification

The error matrix derived from the accuracy assessment of the land cover classification is presented in Table 3. The error matrix revealed a producer accuracy of 98% for forest cover, 97% for logged forest, 85% for settlement and 100% for water.

**Table 3:** Error matrix of the land cover classification.

Land class	Forest	Logged forest	Settlement	Water	Total	Accuracy (%)
Forest	469	10	0	0	479	98
Logged forest	9	429	12	0	450	95
Settlement	0	2	67	0	69	97
Water	0	0	0	10	10	100
Total	478	441	79	10	1008	
Producer Accuracy (%)	98	97	85	100		
Kappa	0.942					
Overall Accuracy (%)	97					

The Kappa coefficient was 0.942 and the overall accuracy was 97% indicating that the classification process avoided about 97% of the errors generated from the completely random classification compared to the ground truth points.

Four (4) LULC classes were identified and classified in the PRB. The classification results (Table 4) show that uncultivated forest has the biggest land area, preceded by logged forest with settlement and water bodies following in that order.

**Table 4:** Percentage area of various land cover classes in the PRB.

Raster Count	Cover Class	Area (km^2^)	Percentage
12316709	Forest	11,085.04	47.87
11586735	Logged Forest	10,428.06	45.03
1769407	Settlement	1,592.47	6.88
56219	Water	50.60	0.22

### 4.2 Spatial distribution of ET in relation to land cover classes

**Figure 2:**
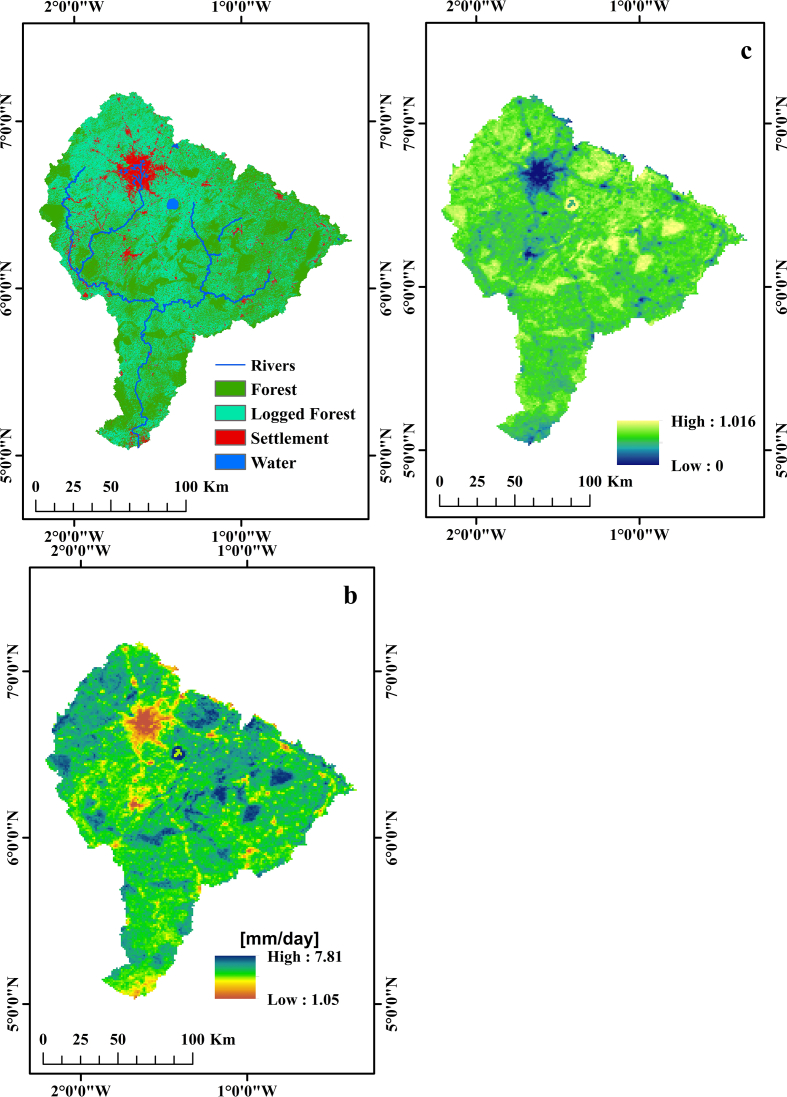
Spatial distributions of ET in relation to land cover classes. a: Land cover classification (2018), b: Daily ET distributions (mm/day), c: Evaporative fraction.

**Figure 3:**
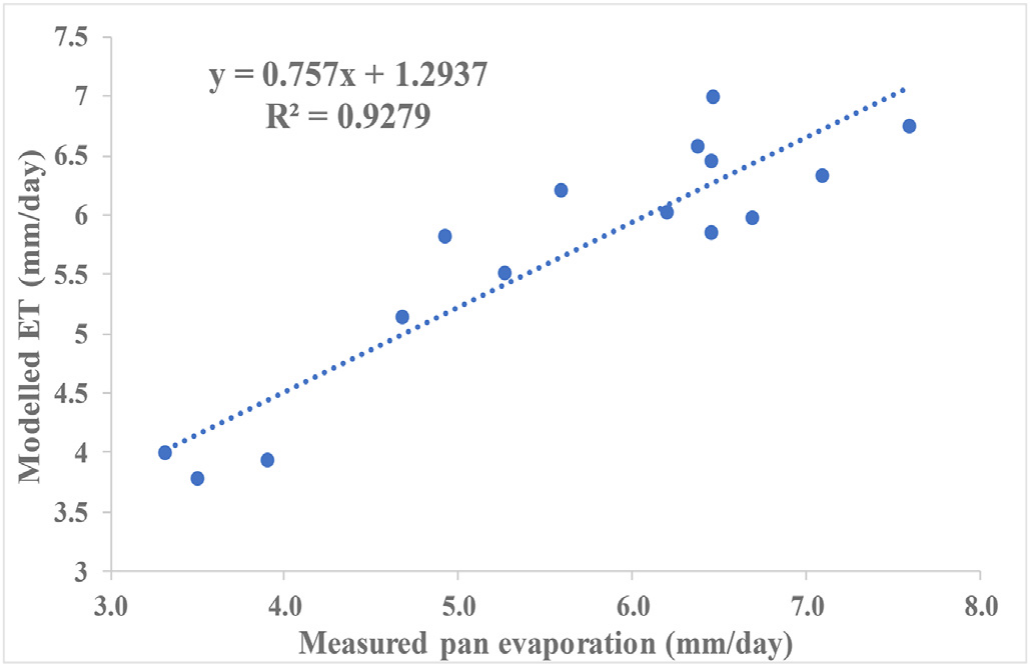
Linear regression model between modelled ET and field measured pan data.

The spatial distribution of daily ET and their corresponding evaporative fraction (ET_rF_) of the atmosphere in relation to land cover classes are presented in Figure 2 and Table 5. The estimated ET ranged from 2.05 to 7.81 mm/day with an overall spatial mean value of 5.63 mm/day. Water body and uncultivated forest(Figure 3) recorded high ET rates ((5.10–7.81) mm/day) compared to logged forest ((4.80–7.51) mm/day) and settlement ((2.05–5.10) mm/day).

**Table 5:** Evaporative fraction and daily ET ranges for various land cover classes within PRB.

No	Land cover classes	ET (mm/day)	ETr_F_ [-]
1	Water body	5.51–7.81	0.29–1.05
2	Forest patches	5.10–7.71	0.29–1.05
3	Logged forest	4.80–7.51	0.26–0.96
4	Settlement	2.05–5.10	0.00–0.50

ET rate observed in relation to the LULC indicates that it varies with Land cover types. Uncultivated forest and water bodies record high ET while settlement and bare landscapes record low ET. These variation points to the fact that LULC types influence the magnitude and spatial distribution in ET. These rates are in line with studies by Ayad et al. (2016) at Tatra mountains in southern Poland; Sun et al. (2011) in Shandong and Jiangsu provinces, China and Opoku-Duah et al. (2008) in the savannah region of West Africa. The high ET rates observed in uncultivated forest and water bodies are attributed to the large volumes of water stored in these LULC types to enhance both evaporation and transpiration (Sett et al., 2018). In contrast, settlement and bare landscapes observed low ET rates ranging from 2 - 5 mm/day. This is attributed to the lack of moisture over these land cover types to facilitate the phase change of latent heat of vaporisation. Similar patterns were found by Bonemberger et al. (2018) and Ayad et al. (2016), who used Landsat 8 images and SEBAL model to estimate radiative fluxes and daily ET distribution. Thus, it is evident that ET rates show a wide range of variation due to the heterogeneous nature of the various land cover classes. This outcome match with other research findings by Dinpashoh et al. (2011); Zhang et al. (2011); and Gao and Zhang (2006).

Linear regression model was applied to quantify the strength of the relationship between the ET distributions of the SEBAL model and that of observed field measurement. The linear regression analysis (Figure 3) showed good fit with slope of 0.76 and R^2^ of 0.93. The outcome revealed that 93 % of the variations in observed field measurement of ET fitted perfectly well with ET distributions generated by the SEBAL model. Thus, indicating that the SEBAL model has a very high potential of estimating the spatial distribution of ET within the study area with high level of accuracy.

### 4.3 Spatial distribution of ET in relation to climatic factors

**Figure 4:**
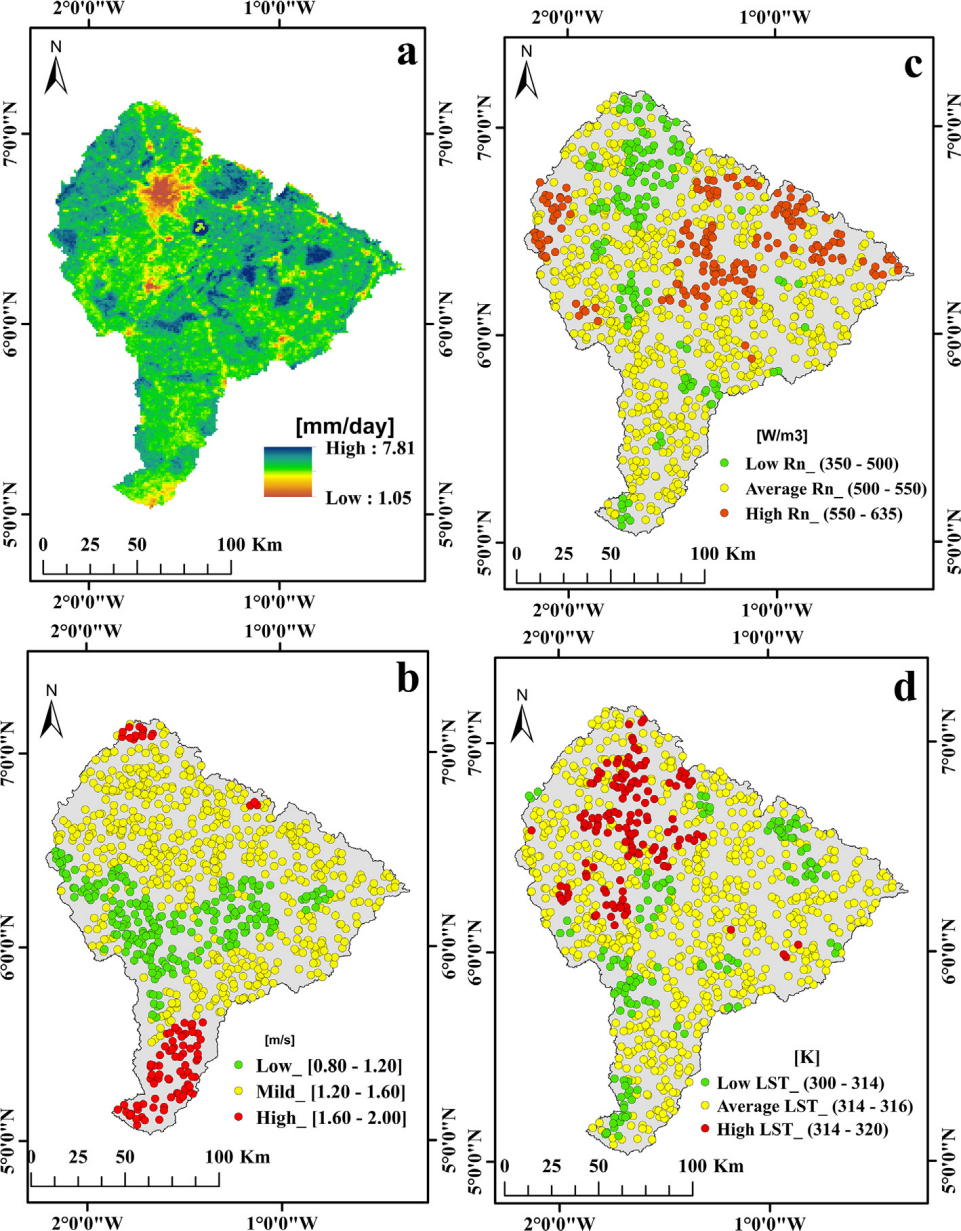
Spatial distribution of major climatic factors and their relation with ET distribution. a: Daily ET estimates (mm/day), b: Wind speed (m/s), c: Net radiation (W/m^2^), d: Land surface temperature (K).

The effect of temperature, wind speed and solar radiation on spatial distribution of ET within the PRB is also presented in Figure 4. According to Li et al. (2018) and Ayad et al. (2016), high temperature with corresponding high solar radiation is associated with high ET as this was observed at the upper western part of the basin (314–320 K). Average temperature (310–314 K) and averagely high solar radiation (500–550 W/m^2^) observed around water bodies together with average wind speed also resulted in average to high ET rates (5.50–6.75 mm/day). At the central to the eastern part of the basin (where both uncultivated and logged forest exist), ET rates were equally high (5.10–7.71 mm/day). This can be explained by the high solar radiation (550–635 W/m^2^), moderately average to high temperature (314–316 K) and mild wind speed (0.80–1.60 m/s) in these areas. On the other hand, low wind speed (0.80–1.20 m/s), low to averagely high temperature (300–316 K) and widely varying solar radiation existing within the central part of the basin (where logged forest is prevalent) also resulted in moderately average to high ET rates (5.10–7.81 mm/day).

While at the southern part of the basin (where forest patches, logged forest, traces of water bodies and clusters of settlement and bare landscape exist), ET rates ranged between 4.80 mm/day to 7.70 mm/day representing average to high ET rates. The estimated high temperature in settlements and bare landscapes, coupled with widely varying solar radiation and wind speed resulting in low ET are in-line with studies by Li et al. (2018); Ayad et al. (2016). Thus spatially, areas with high temperature and high solar radiation experiences high ET (especially, when forest and water bodies occur), while low wind speed, low to average temperature and solar radiation areas experience low ET. Thus, the distribution of ET was found to vary depending on the land use type and climatic variables as established by Yonggwan and Seongjoon (2016).

### 4.4 Spatial distribution of ET in relation to energy balance components

**Figure 5:**
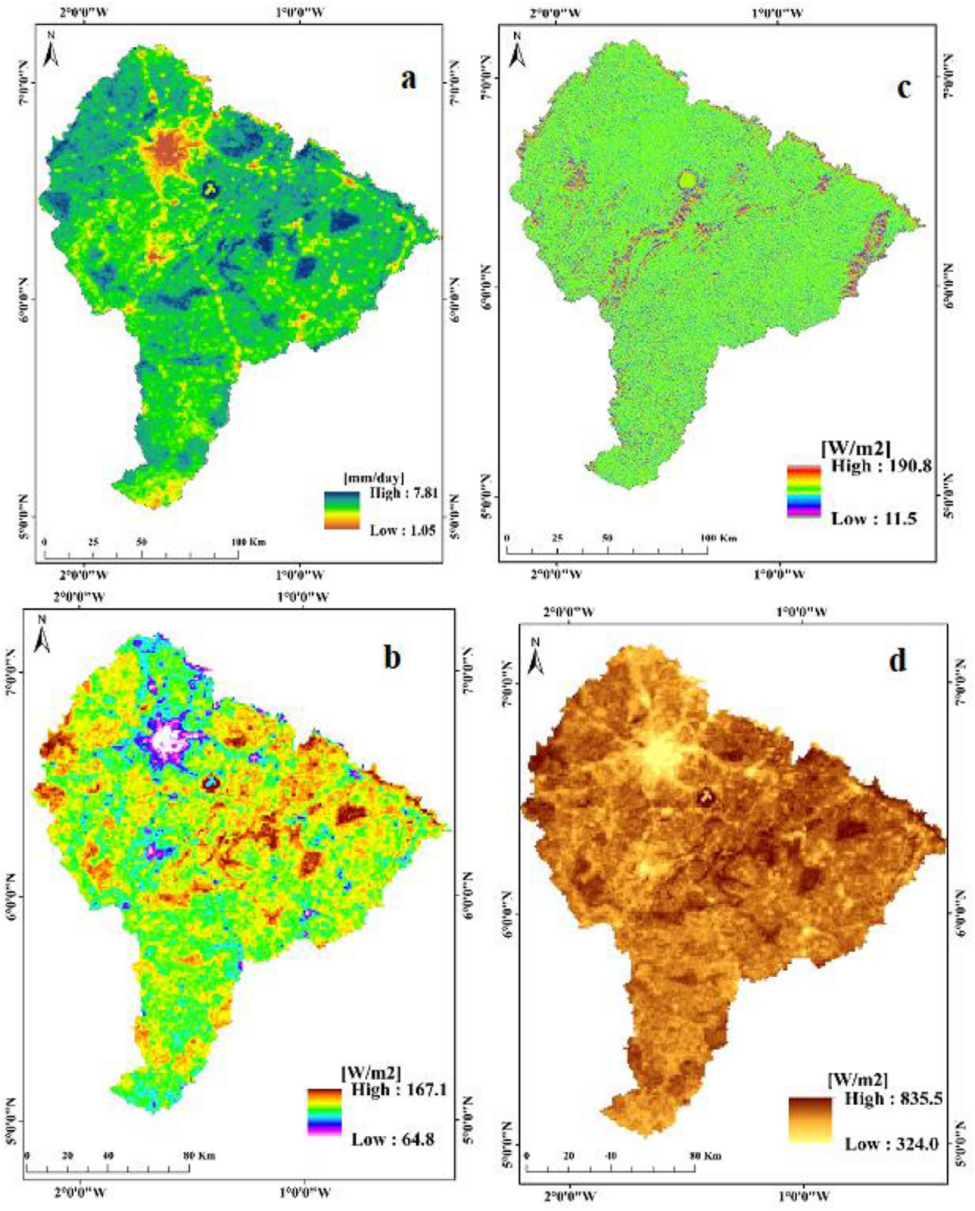
Spatial distribution of the energy balance components. a: Daily ET estimates (mm/day), b: Soil heat flux (W/m^2^), c: Sensible heat flux (W/m^2^), d: Net radiation flux (W/m^2^).

Investigations into the influence of the energy balance components on spatial distribution of ET (Figure 5) revealed that, net radiation is very high (780–835) W/m^2^)) in uncultivated and logged forest (720–812) W/m^2^)) than settlement and bare landscape (324–365) W/m^2^)). This outcome is in line with the findings of Li and Zhao (2010a, b), who used the SEBAL model to compute the ET rate for the middle reach of the Heihe River Basin in China. This difference in net radiation is mainly influenced by difference in reflective properties of the various LULC surfaces. Thus, the uncultivated forest with low reflective properties absorbs more heat energy than bare lands which has high reflective properties (Sett et al., 2018). Similarly, soil heat flux is also high in uncultivated and logged forest than settlement and bare landscape. The greater the soil heat flux the greater the conduction of heat in the soil. Uncultivated and logged forest which have wet soil have high thermal conductivity than settlement and dry bare landscapes.

Thus, the soil heat flux which is dependent on the amount of moisture present in the soil varies spatially in relation to the heterogeneous nature of the land cover types. There was high evaporation rate in uncultivated and logged forest (as stated earlier) which also led to high latent heat loss. But for settlement, the latent heat flux was lower with a correspondingly lower sensible heat flux which agrees with the findings of Sett et al. (2018). Although, in contrast, significant increases in ET were sporadically distributed within the southern part of the basin. It was also observed that high latent heat is driven by cooling of land surface while low latent heat is driven by heating of land surface. The computed soil heat flux registered its peak value of 167.1 W/m^2^ in uncultivated and logged forest while a low value of 64.8 W/m^2^ was recorded over settlement and bare landscape as shown in Figure 5 b. It is clear that the spatial distribution of ET has direct relation with soil heat, sensible heat and the net radiation flux of the surface energy budget equation.

## 5 Conclusions and recommendations

In this study, 3 cloud-free Landsat 8 (OLI/TIR) images from different path and rows were mosaicked and used to model the spatial distribution in ET patterns based on SEBAL model within the PRB. Results show a distinct pixel-wise variation in the pattern of ET with an overall spatial mean value of 5.63 mm/day. Daily ET (mm/day) for water body (5.51–7.81) and uncultivated forest (5.10–7.71) were high while moderately average values were estimated for logged forest (4.80–7.51). In contrast, settlement and bare landscapes observed low (2.05–5.10) daily rates owing to the lack of moisture over these land cover types to facilitate the phase change of latent heat of vaporisation. The low daily ET rates (2.05–5.05 mm/day) observed at the northern part of the basin where settlement and bare landscapes dominate, were attributed to mild wind speed and traces of low solar radiation. Also, high temperature and high solar radiation coupled with mild wind speed observed around water bodies resulted in high ET ranges (5.51–7.81) mm/day. At the upper western, central and eastern parts of the basin where uncultivated and logged forest exist, ET ranges were very high (4.80–7.71) mm/day. This can be explained by the presence of high net solar radiation, average to high temperature and mild wind speed in these areas. On the other hand, low wind speed, low to average temperature and average to high solar radiation existing within the central part of the basin where logged forest dominates also resulted in averagely high ET rates (5.05–7.68 mm/day). At the southern part of the basin where forest patches, logged forest, traces of water bodies and clusters of settlement and bare landscape exist, ET rates were spatially distributed ranging between 2.05 mm/day and 7.69 mm/day. Linear regression analysis showed good fit with slope and r^2^ of 0.76 and 0.93 respectively. This shows that 93 % of the variations in the observed field measurement of ET were well fitted and explained by ET distributions generated by the SEBAL model. This proves that the SEBAL model has a very high potential of estimating the ET distribution within the study area with high level of accuracy and thus will serve as an important tool for planning and management of river basin studies.

## Declarations

### Author contribution statement

J. J. Nsiah: Conceived and designed the experiments; Analyzed and interpreted the data; Wrote the paper.

C. Gyamfi, S. N. Odai: Analyzed and interpreted the data.

G. K. Anornu: Conceived and designed the experiments.

### Funding statement

This work was supported by the Ghana Government and the World Bank under the Africa Centre's of Excellence project (ACE I-P126974-Cr. No. 54230).

### Data availability statement

Data will be made available on request.

### Declaration of interest statement

The authors declare no conflict of interest.

### Additional information

No additional information is available for this paper.
